# Sanitary Condition of Troops

**Published:** 1861

**Authors:** 


### SANITARY CONDITION OF TROOPS.

We publish with pleasure the following excellent directions for the sanitary conduct of the troops in the field, which constitute the substance of a report prepared by Dr. John Ware, of the State Medical Commission, to be communicated to the Massachusetts regiments in active service.

*Directions.*—Soldiers should recollect that in a campaign, where one dies in battle, from three to five die of disease.—You should be on your guard, therefore, more against this than the enemy, and you can do much for yourselves which nobody can do for you.

1. Avoid, especially, all use of ardent spirits. If you will take them,—take them rather *after* fatigue than before.—But tea and coffee are much better. Those who use ardent spirits are always the first to be sick and the most likely to die.

2. Avoid drinking freely of very cold water, especially when hot or fatigued, or directly after meals. Water quenches thirst better when not very cold and sipped in moderate quantities slowly—though less agreeable. At meals, tea, coffee, and chocolate are best. Between meals, the less the better. The safest in hot weather is molasses and water, with ginger, or small beer.

3. Avoid all excesses and irregularities in eating and drinking. Eat sparingly of salt and smoked meats, and make it up by more vegetables, as squash, potatoes, peas, rice, hominy, Indian meal, etc., when you can get them. Eat little between, when you have plenty at meals.

4. Wear flannel all over in all weathers. Have it washed often when you can—when not, have it hung up in the sun. Take every opportunity to do the same by all your clothing, and keep everything about your person dry, especially when it is cold.

5. Do not sit, and especially do not sleep upon the ground, even in hot weather. Spread your blanket upon hay, straw, shavings, brush-wood, or anything of the kind. If you sleep in the day, have some extra covering over you.

6. Sleep as much as you can, and whenever you can. It is better to sleep too warm than too cold.

7. Recollect that cold and dampness are great breeders of disease. Have a fire to sit around whenever you can, especially in the evening and after rain, and take care to dry everything in and about your persons and tents.

8. Take every opportunity of washing the whole body with soap and water. Rub well afterwards. If you bathe, remain in the water but a little while.

9. If disease begins to prevail, wear a wide bandage of flannel around the bowels.

10. Keep in the open air, but not directly exposed to a hot sun. When obliged to do this, a thin, light, white covering over the head and neck, in the form of a cap with a cape, is a good protection.

11. Wear shoes with very thick soles, and keep them dry. When on the march, rubbing the feet, after washing, with oil, fat or tallow, protects against foot-sores.—*Boston Medical Journal.*

